# The Role of Friendship in Mediating and Moderating the Relationship Between Exposure to Gendered Racism and Mental Health among Young Women of Color

**DOI:** 10.1007/s10964-024-02130-3

**Published:** 2025-01-02

**Authors:** Xiangyu Tao, Celia B. Fisher

**Affiliations:** 1 Department of Psychiatry, Rutgers Addiction Research Center, Rutgers Robert Wood Johnson Medical School, 671 Hoes Lane West, Piscataway, NJ USA; 2 Department of Psychology, Fordham University, 441 East Fordham Road, Dealy Hall, Bronx, NY USA; 3 Center for Ethics Education, Fordham University, 441 East Fordham Road, Dealy Hall, Bronx, NY USA

**Keywords:** Gendered Racism, Friendship, Discrimination, Social Media, Mental Health, Co-Rumination

## Abstract

Young women of color frequently face discrimination, reflecting the intersecting societal influences of sexism and racism. Although friendships play a significant role in women’s lives, there is a lack of research on the role of friendships in navigating exposure to gendered racial discrimination (in-person and social media) and associated mental health. This study investigated the extent to which the content of friendship conversations (i.e., co-rumination against gendered racism, socializing messages related to gendered racial pride and empowerment and oppression awareness) and perceived friendship intimacy and support mediated or moderated the positive association between exposure to gendered racism and mental health. Co-rumination was tested as a mediator, while the other variables were examined as moderators. Online survey data were collected from 339 cisgender women aged 18–24 (*M age* = 20.90, *SD* = 1.96; 32.74% Asian, 33.92% Black, and 33.33% Hispanic or Latina; 68.14% identified as straight or heterosexual). Participants described friendship communications and perceived intimacy and support with a same gender and race close friend. Exposure to gendered racial discrimination was significantly associated with depressive and anxiety symptoms and substance use as a coping mechanism. Co-rumination about gendered racism was positively correlated with depressive and anxiety symptoms. Conversely, pride and empowerment socialization was negatively associated with substance use for coping, while oppression awareness socialization was positively correlated with depressive and anxiety symptoms. Structural Equation Modeling Analyses indicated that co-rumination partially mediated the relationship between gendered racism exposure and anxiety symptoms, with other friendship indices not moderating these associations. These findings highlight co-rumination about gendered racism as a risk factor for young women of color and underscore the importance of exploring how the multifaceted nature of friendships is associated with exposure to gendered racism and mental health.

## Introduction

Gendered racism reflects the unique intersectional vulnerability experienced by women of color rooted in a system of oppression at the intersection of sexism and racism embedded within institutional policies, societal structures, and cultural norms (Crenshaw, 1989; Essed, 1991). Gendered racial discrimination, as part of this broader system, occurs when women of color personally or indirectly experience unequal or disadvantageous treatment based on their intersecting gender and racial identities (Bowleg, 2012; Cole, 2009). Emerging adulthood, particularly in the earlier years (ages 18–24), is a critical developmental stage marked by increased autonomy, identity exploration, and shifts in social roles as young adults navigate independence, often for the first time, by moving away from home (Arnett, 2000). During this period, friendships become primary sources of social support, replacing reliance on parental guidance and serving as a key influence on psychological well-being (Veenstra & Laninga-Wijnen, 2021). Young adults are also most active online among adult age groups, with 97% reporting regular internet use in 2023 (Gelles-Watnick, 2024), and 93% reporting social media use (Gottfried, 2024). This extensive digital engagement may increase young women of color’s risk of experiencing online gendered racial discrimination and influence their friendship communications regarding such experiences. Although research on friendships in response to gendered racial discrimination remains limited, prior studies highlight the protective role of friendships in mitigating mental health challenges arising from identity-threatening stressors, i.e., experiences that challenge central aspects of self-identity, such as one’s racial or gender identity. These stressors include racial discrimination (Cénat et al., 2024), as well as other challenges like relationship infidelity or health diagnoses, which may not necessarily stem from discrimination but still disrupt one’s sense of self (Davis & High, 2019). This study aimed to extend understanding of the role of close friendships in the lives of young women of color by examining associations among friendship communications specific to gendered racism and the relationship between exposure to in-person and social media gendered racial discrimination and mental health. Throughout this article, the term “gendered racial discrimination” is used to refer to experiences of discrimination based on both race and ethnicity, acknowledging that both identities may contribute to intersecting forms of oppression.

Aligned with the minority stress theoretical framework (Frost & Meyer, 2023), the adverse impact of in-person racial discrimination on mental health among young adults of color has long been supported, with such experiences often disrupting daily routines and directly affecting engagement in immediate environments (Le & Iwamoto, 2019; Neblett et al., 2016). Social media presents an additional context for personally directed racial discrimination and vicarious stress by extending audiences and amplifying the frequency of exposure to racist posts (Nesi et al., 2018). While social media and in-person exposures to discrimination differ in context and frequency, both are interconnected phenomenological experiences that uniquely and collectively impact mental health outcomes (Fisher et al., in press). Accordingly, recent studies show that online (social media) exposure to racial discrimination, both individual (directed at the person) and vicarious (directed at other people of color), is associated with increased mental health risks among youth and young adults of color (Portillo et al., 2022; Tao & Fisher, 2022), with vicarious discrimination being particularly prevalent in online settings (El-Tohamy et al., 2024). Similarly, exposure to social media individual and vicarious gender discrimination is associated with mental health risks among young women (Lewis, 2018; Sobieraj, 2018).

Emerging research indicates experiences of discrimination directed at the intersection of gender and racial social positions increase mental health risks among Asian (Forbes et al., 2023), Black (M. K. Jones et al., 2022; M. S. Jones et al., 2021), and Hispanic or Latina women (M. K. Jones & Briones, 2022). These studies have primarily focused on the unique experiences of gendered racial discrimination within specific racial or ethnic groups, which may vary depending on institutionalized gendered racial stereotypes. Meanwhile, it is equally important to recognize that systemic injustices related to gender and race create commonalities in the discrimination experienced by women of color in the U.S. (Collins & Bilge, 2020). Acknowledging both the unique and shared aspects of these experiences provides a more comprehensive understanding of gendered racism and offers opportunities for solidarity among groups.

High quality friendships, characterized by perceived intimacy and support, have been associated with better mental health among young women of color. For example, an early study indicated how general social support acts as a protective mechanism associated with better mental health and buffering against the adverse effects of stress (Turner, 1999) and a recent review of 64 articles underscored how higher friendship quality support young adults’ mental health (Navaneetham & Kanth, 2022). Previous research also highlights how friendships can alleviate stressors that challenge one’s sense of identity arising from relationship infidelity or health diagnoses (Davis & High, 2019). Importantly, friendships have been found to protect against identity-based discrimination. For example, friends’ social support has been shown to buffer the negative impact of racial discrimination on self-esteem among Black Canadians (Cénat et al., 2024). Compared to men, women are more likely to establish friendships through discussion and self-disclosure, potentially enhancing the mental health benefits of these supportive bonds (Pearce et al., 2021).

Less is known about young women of color’s friendship conversations related to their experiences of gendered racism. For example, women exposed to gendered racism may co-ruminate about these experiences with friends, defined as excessively discussing, rehashing, and speculating about these experiences with a focus on negative affect (Rose, 2002). While rumination is an individual cognitive process marked by a repetitive focus on distress without interpersonal input (Nolen-Hoeksema et al., 2008; Watkins & Roberts, 2020), co-rumination occurs in a relational context, involving shared emotional intensity and detailed revisiting of problems. Co-rumination may initially provide validation and social connection, yet its repetitive and emotion-focused nature often amplifies mental health symptoms rather than fostering problem resolution. This dual nature of co-rumination has been observed in studies linking it to higher friendship quality, risk for depressive and anxiety symptoms among young women (Spendelow et al., 2017), and mental health symptoms during the COVID-19 pandemic (Stone & Veksler, 2022; Zhou et al., 2020). One study specifically focused on racism found co-rumination partially mediated the association between exposure to in-person racial discrimination and mental health symptoms among Black young adults (Hacker et al., 2016). Social media may also amplify women’s exposure to both gendered racial discrimination and co-rumination with friends by facilitating constant communication with both friends and strangers and easy access to information regardless of physical distance (Angelini et al., 2022; Are, 2020).

Friendships among young women of color also involve gendered racial socialization that promotes empowerment or emphasizes the oppressive nature of gendered racism. Racial socialization originally referred to parent-child communication promoting racial or ethnic pride and empowerment while raising awareness of oppression (Fisher et al., 2000; Hughes & Johnson, 2001; Stevenson, 1994). More recently, peer-based racial or ethnic socialization has been studied in young women. For example, positive mental health was associated with peer-based gendered racial empowerment among Asian young adult women (Ahn et al., 2022). Friends’ racial empowerment messaging was also found to mitigate the impact of discrimination on mental health for Black young adults (Su et al., 2020). However, gendered racial oppression discussions may exacerbate mental health risk by reminding women of traumatic racial experiences. For instance, recent studies with Black adolescent girls indicated discussions about discrimination were linked to higher levels of depressive symptoms (Stokes et al., 2020; Winchester et al., 2022).

Given the complexity of young women of color’s experiences navigating gendered racism, several individual characteristics may influence associations between gendered racism and mental health. Racial or ethnic identity (REI), defined as the thoughts, feelings, and attitudes related to one’s racial or ethnic group membership, may impact how young adults of color experience and cope with discrimination (Yip, 2018). Centrality, or the extent to which one’s racial or ethnic identity is a core part of their self-concept, may shape one’s stress responses to discrimination, while commitment, reflecting a strong and positive connection to one’s racial or ethnic identity, may be associated with better mental health (Yip et al., 2019). Mode of friendship communication (i.e., percent of time spent with the same friend in-person versus online), may also impact the relationship between discrimination and social support (Nesi et al., 2018; Tao & Fisher, 2023, 2022). Additionally, mental health can vary based on demographic factors such as sexual orientation (Paley, 2022), race and ethnic group membership, and socioeconomic status (Neblett et al., 2016).

## The Current Study

Despite growing recognition of the negative impact of gendered racial discrimination on mental health, there is a paucity of research examining how these experiences intersect with friendship dynamics among young women of color. Drawing upon the intersectionality and minority stress theoretical frameworks, this study explored the association between exposure to in-person and social media gendered racial discrimination and mental health (i.e., depressive and anxiety symptoms and substance use as a coping mechanism against discrimination) among Asian, Black, and Hispanic or Latina young adult women, and examined how same gender and race friendship communications mediate or moderate these associations. Several hypotheses were tested. The first 2 hypotheses predicted that exposure to gendered racial discrimination and friendship communications characterized by co-rumination about gendered racism and emphasizing gendered racial oppression would be positively associated with depressive and anxiety symptoms and substance use as coping. A third hypothesis tested was that communications about gender and race emphasizing pride and empowerment and those indicating friendship intimacy and support would be negatively associated with these mental health symptoms and behaviors. In addition, it was predicted that co-rumination would partially explain the positive association between exposure to discrimination and depressive and anxiety symptoms and substance use as coping. It was also anticipated that pride and empowerment socialization and friendship intimacy and support would mitigate the positive associations between exposure to discrimination and mental health symptoms, whereas oppression awareness socialization would exacerbate such associations. Building upon these hypotheses, the final hypothesis tested whether the mediating influence of co-rumination on the association between exposure to discrimination and depressive and anxiety symptoms and substance use as coping would be moderated by socialization and friendship intimacy and support. REI development, the mode of friendship communication (percentages in-person versus online), race and ethnicity, sexual identity, and socioeconomic status were considered as covariates. To further illuminate quantitative findings, participants were also asked an open-ended question related to participants’ spontaneous descriptions of racial or ethnic group specific gendered racial stereotypes and the ways these may shape experiences of discrimination.

## Methods

### Participants and Recruitment

To ensure racial and ethnic diversity, the study aimed to recruit 100 participants from each of Asian, Black, and Hispanic or Latina groups. This sample size was chosen to achieve a power level greater than 0.90 for a structural equation model (SEM) analysis with more than 100 degrees of freedom and an expected root mean square error of approximation (RMSEA) of 0.05 (MacCallum et al., 1996). Eligible participants were aged 18–24, assigned female at birth, identified as a cisgender woman, self-identified as Asian, Black, or Hispanic or Latina, resided in the U.S., and had English competency at 8^th^ grade. The final screening criteria required exposure to gendered racism in the past month and having a close same-gender, same-race and ethnicity friend with whom they felt comfortable discussing racial issues. Specifically, participants were asked, “Gendered racism is personal experiences of harassment or discrimination based on both one’s race and gender or witnessing this type of harassment or discrimination of others. Gendered racism can occur in person or online. To what extent have you experienced gendered racism in the past month?” Response options ranged from “Never” to “Daily and almost Daily”. For the friendship criterion, participants were asked to check all that applied to describe their friendships, including 4 statements: “I have a close friend who is a woman (or man) of my race/ethnicity (or from a different racial or ethnic group), with whom I frequently discuss my experiences, thoughts, and feelings about racism.” Only participants who selected the option indicating they had a close friendship with a woman of their own race/ethnicity were included in the study. Before data collection, 1-hour discussions were conducted with 8 college freshman young women of color (mean age of 19) serving as advisory board members including e Asian, 4 Black, and 1 Hispanic or Latina women. During discussions, advisory board members provided feedback on the survey design to help ensure that the study was age appropriate, culturally sensitive and that the questions were relevant and understandable to the target population.

Data were collected between August and September 2023 through Qualtrics Marketplace©, an aggregator of survey panel websites to recruit individuals who have signed up to take paid surveys across multiple sites. Qualtrics sent emails to individuals fitting the age (18–24), gender (female), and race/ethnicity criteria, inviting them to participate in an anonymous online survey. Participants who met the inclusion criteria and provided informed consent were given a unique identification number and access to the main survey. Participants could quit the survey at any time by closing the survey window, and their data were not included in analyses. Participants’ identities were unknown to the investigator. To ensure data integrity, automated data validation protocols were implemented, including speed checks that excluded any responses completed in less than half of the median response time, and two attention check questions. Researchers conducted manual data verification to confirm the consistency of reported ages with dates of birth. Participants who provided valid responses to the survey were compensated with the pre-agreed reward points from Qualtrics, which could be exchanged for gift cards. In total, 2,942 individuals took the screening survey, 563 met the eligibility requirements, and 494 provided consent. A total of 341 participants completed the survey and passed the automated validation checks, and 339 passed the manual check, forming the final sample for analysis, included 111 Asian, 115 Black, and 113 Hispanic or Latina participants, with an average survey completion time of 21.43 minutes (SD = 15.38). Approval for all study procedures was obtained from the Fordham University IRB.

### Measures

#### Demographic Items

Participants self-reported age, gender, race and ethnicity, sex at birth, sexual identity, and social media use frequency (days per week and hours per day). Socioeconomic background was assessed through 3 variables: (1) Highest education level, (2) Financial security status with response options: (a) I can’t make ends meet, (b) I have just enough money, and (c) I am financially comfortable, and (3) The participants’ perceptions of their level of social inequality measured by the adapted MacArthur Scale of Subjective Social Status (Adler et al., 2000), a single-item measure using a ladder picture is used to represent the social hierarchy of American society. Participants rate their socioeconomic status by placing them on the ladder (“Please rate from 1–10 that best represents where you think you stand on the ladder compared to people like you”). Participants also reported their designated friend’s race and ethnicity and age.

#### Exposure to Gendered Racial Discrimination

The scale focused on women’s self-reported experiences of gendered racial discrimination consistent with theory and research indicating that perceived discrimination, irrespective of its objective basis, is closely linked to higher mental health risk (Fisher et al., in press). Four subscales measured the past month’s experiences with in-person and social media individual and vicarious gendered racial discrimination. The in-person individual discrimination was assessed using a 9-item scale adapted from the Everyday Discrimination Scale (Williams et al., 1997) modified for gender and race and ethnicity (e.g., “I have been treated with less courtesy because I am a woman of my race/ethnicity”). Vicarious in-person discrimination utilized five items from the Vicarious Racism Scale (Martz et al., 2019) and the adapted Online Victimization Scale (Tynes et al., 2010), such as “I have seen women of my race/ethnicity treated unfairly in public.” Individual and vicarious social media discrimination were assessed by the adapted Online Victimization Scale. “Online” was substituted with “on social media” for all items, and “people/person” was replaced with “woman/women” in each statement. Respondents rated experiences on a 5-point scale from (0) *Never* to (4) *Almost daily*. In the current study, Cronbach’s α = 0.95 for the total scale, with each subscale ranging from 0.87 to 0.92. To ensure that the discrimination scale was interpreted consistently across Asian, Black, and Hispanic or Latina participants, measurement invariance testing were conducted. The baseline model results indicated that the factor structure was consistent across groups (χ² = 909.19, df = 549, RMSEA = 0.077, CFI = 0.97, TLI = 0.97). Threshold invariance was established (Δχ² = 91.66, Δdf = 84, *p* = 0.27; RMSEA = 0.071, CFI = 0.97, TLI = 0.97), demonstrating equivalence in item thresholds. Full invariance of thresholds and loadings was also confirmed (Δχ² = 32.89, Δdf = 34, *p* = 0.52; RMSEA = 0.067, CFI = 0.97, TLI = 0.97), indicating consistent interpretation of item loadings across groups.

To better understand how gendered racism stereotypes intersect with discrimination experiences, participants were asked to reflect on the statement: “Please discuss in what ways stereotypes about women of your race or ethnicity may increase or decrease the exposure to discrimination?”.

#### Friendship Experiences

Participants reported the frequency of conversations with their friends about race-related issues (i.e., “In the past month, how frequently have you and the close friend you just nominated discussed issues related to race?” with response options ranging from (0) Never to (7) Seven days a week. Responding to a sliding bar ranging from 0 to 100, respondents indicated the percentage of time spent discussing race-related issues online (i.e., “You may communicate with your friends online or in person. What percent of your time discussing issues related to race with your friend was online in the past month?”). Additionally, a 0 – 100% sliding bar item was included for 3 scales assessing the amount of time spent on different types of friendship communications online and in person: gendered racism co-rumination, pride and empowerment socialization, and oppression awareness socialization. These ratings served as single item descriptive variables and potential covariates to account for the mode of communication in our analysis. Except for these 0–100% rating questions, the friendship scales do not differentiate between in-person and social media interactions. This approach was guided by recommendations from the community advisory board, which noted that most young women’s friendships involve a blend of in-person and social media communication.

##### Gendered Racism Co-Rumination

To assess the extent to which participants co-ruminate with their close friends about in-person and online gendered racism, the study adapted the 27-item Co-Rumination Questionnaire (CRQ.; Rose, 2002) by replacing “problem” with “gendered racism” (e.g., “We often spend time together talking about gendered racism that my friend or I have exposure to”; “When we see each other, if one of us has exposure to gendered racism, we will talk about the problem even if we had planned to do something else together”). Participants were shown the following prompt: “Gendered racism may take many forms and include a wide range of experiences. Exposure to gendered racism include not only your personal experiences that you felt were discrimination related to being a woman of color, but also viewing other women of color being treated differently. Thinking about that close friend you nominated, when you are with her…” Response options ranged from (0) Never to (3) Always. In the current study, Cronbach’s α = 0.95.

##### Gendered Racial Socialization

Participants were asked to evaluate the frequency of messages exchanged with friends about gendered racial socialization. Specifically, the 18 items comprising the Gendered Racial or ethnic Pride and Empowerment subscale were intended to capture the bidirectional nature of communication. The stem sentence for this measure is: “How often do you and your close friend who you just nominated communicate these messages…”. Responses ranged from (0) Never to (4) Always. Sixteen items (e.g., “I should be proud to be a woman of my race/ethnicity”) were adapted from the Gendered Racial-Ethnic Socialization Scale for Black Women (GRESS-BW; Brown et al., 2017), and 1 each from the Asian American Parental Racial-Ethnic Socialization Scale (AAPRIS; i.e., “It’s important to spend time with women of my race/ethnicity”; Juang et al., 2016), and the Latina Immigrant Family Socialization Scale (LIFSS; i.e., “the contribution of women of my race/ethnicity to the United States”; Ayón, 2018). The 8-item Gendered Racial or ethnic Oppression Awareness subscale combined six items from the GRESS-BW Oppression Awareness and the Gendered Racial Hardship subscale (e.g., “There are more opportunities for White women, so as a woman of my race/ethnicity, I have to work twice as hard”) and two items from the AAPRIS (e.g., “People may try to take advantage of me because I am a woman of my race/ethnicity”). In the current study, Cronbach’s α = 0.94 and 0.83 for the Pride and Empowerment and Oppression Awareness subscales.

##### Perceived Intimacy and Support

To assess friendship intimacy and support, 10 items were adapted from the short version of the McGill Friendship Questionnaire – Friend’ s Function subscale with 5-items taken from the Help subscale (e.g., “helps me when I need it”) and 5-item from the Intimacy subscale (e.g., “knows when I’m upset”) (Mendelson & Aboud, 1999). Response options ranged from (0) Never to (4) Always. In the current study, Cronbach’s α = 0.77 and 0.82 for the intimacy and support subscales.

#### Mental Health

Depressive symptoms over the past month were assessed using the 9-item Patient Health Questionnaire (PHQ-9; e.g., “Feeling down, depressed or hopeless.”; Kroenke et al., 2001) using a 4-point scale from (0) Not at all to (3) Nearly every day. Anxiety symptoms over the past month were measured with the 7-item Generalized Anxiety Disorder Screener (GAD-7; e.g., “Being so restless that it is hard to sit still”; Spitzer et al., 2006) using the same 4-point scale. For both scales, composite scores were computed by the sum of item responses. Cronbach’s α = 0.89 and 0.91 for the PHQ-9 and GAD-7 in the current study. The 5-item Drug and Alcohol Use subscale of the Coping with Discrimination Scale (Wei et al., 2010) was adapted to assess the extent to which women used substances to cope with negative experiences (Moody et al., 2022; Szymanski & Lewis, 2016). Participants were asked to, “Read each of the following statements and indicate how often you have been using each coping strategy to deal with gendered racism” (e.g., “I use drugs or alcohol to take my mind off things.”). Items were rated on a 6-point scale ranging from (1) never like me to (6) always like me. The sum of the item scores was used to create a final score. Cronbach’s α = 0.86 in the current study.

#### Covariates

The survey included measuring several individual characteristics that may influence associations between gendered racism and mental health, including demographic items (i.e., race and ethnicity, sexual identity, financial security, and self-reported social status) and percent of time spent with friends in-person versus online.

##### Racial or Ethnic Identity (REI) Development

REI development was assessed using two instruments: the revised 12-item Multigroup Ethnic Identity Measure (MEIM; Phinney, 1992) and the 8-item Centrality scale from the Revised Multidimensional Inventory of Black Identity (MIBI; Sellers et al., 1997). The MEIM includes items such as “I have spent time trying to find out more about my ethnic group, such as its history, traditions, and customs” (Exploration) and “I have a clear sense of my ethnic background and what it means for me” (Commitment), and the MIBI Centrality scale includes items such as “Overall, my cultural or ethnic background has very little to do with how I feel about myself.” Participants rated these items on a scale of (1) strongly disagree to (7) strongly agree. Scores for each component were calculated as the average of respective subscale items. In the current study, Cronbach’s α = 0.91, 0.80, and 0.74 for Commitment, Exploration, and Centrality subscales.

### Data Analysis

Data analyses were conducted using IBM SPSS Statistics (Version 28.0) and Mplus (Version 8.3). Descriptive statistics was first calculated, followed by Chi-square and Analysis of Variance (ANOVA) tests with Tukey’s post hoc comparison to examine racial differences in demographics and studied variables. To examine the first two hypotheses on associations between exposure to discrimination and friendship characteristics with mental health, bivariate Pearson’s correlations were tested, adjusting for significance with Bonferroni correction at α = 0.05. To examine the mediation hypothesis (i.e. whether co-rumination mediated the association between discrimination and mental health), SEM analyses were adopted with the Weighted Least Squares Mean and Variance Adjusted (WLSMV) estimation based on the ordinal nature of data. For the moderation hypothesis (i.e. friendship socialization and quality moderated the associations between discrimination and mental health), the Latent Moderated Structural Equations (LMS) approach was adopted (Klein & Moosbrugger, 2000). Moderation analyses within a mediation SEM framework were adopted for the final moderated mediation hypothesis. The same covariates were included in all SEM analyses and were selected based on prior research and theoretical relevance to mental health indices. In cases where high correlations between multiple covariates might lead to multicollinearity, covariates that demonstrated lowest covariance with other variables were prioritized. For sensitivity analyses, to examine potential alternative associations between co-rumination and mental health symptoms (e.g., women with more mental health symptoms may be more likely to co-ruminate with friends as a coping strategy for discrimination), the mediating roles of depressive and anxiety symptoms and substance use as coping in the association between discrimination and co-rumination were tested. The sensitivity analysis ensures the robustness of the main research question findings and accounts for alternate explanatory pathways. The goodness of fit indices for SEM analyses included the comparative fit index (CFI), Tucker-Lewis Index (TLI), and the Root Mean Square Error of Approximation (RMSEA). A fit of > 0.90 for the CFI and TLI and < 0.06 for RMSEA indicate adequate fit (Hu & Bentler, 1999). A bias-corrected bootstrapping approach was further utilized for the SEM analyses to test the indirect effects for statistical significance with 1000 resamples drawn to estimate standard errors of the indirect effect and 95% confidence intervals (CIs).

Qualitative analysis was conducted on the open-ended question to capture participants’ descriptions of gendered racial or ethnic stereotypes. Using an inductive thematic approach (Macqueen et al., 1998), two team members independently reviewed the responses, noting commonalities and distinctions in the stereotypes described. Through this process, common and unique gendered racial stereotypes across participants from the 3 racial and ethnic groups were identified.

### Missing Data

Items on sexual identity and education level provided participants the option to decline to answer or indicate that they do not know the answer. However, none of the participants endorsed any of those options, thus there was no missing data in the current study. For the “I am not sure” in the sexual orientation question, the four participants who endorsed this option were categorized as questioning consistent with theoretical frameworks on developmental stages of sexual identity (Cyrus & Morrison, 2019). Chi-square tests were conducted to examine the demographic differences between participants who dropped out (8.2%, *n* = 41) with those who completed the survey, and yielded no significant differences on all screener measures, including age, X^2^(6) = 1.38, *p* = 0.97, race/ethnicity, X^2^ (2) = 3.17, *p* = 0.20, sexual identity, X^2^ (7) = 3.36, *p* = 0.85, social media use frequency per week, X^2^ (7) = 11.11, *p* = 0.13, social media use frequency per day, X^2^ (10) = 8.5, *p* = 0.58, frequency exposure to gendered racism, X^2^ (3) = 1.18, *p* = 0.76, and frequency discussing racial issues with friends they nominated, X^2^(5) = 2.51, *p* = 0.77.

## Results

### Demographic Data

Frequency, percentages, and Chi-square test results of demographic data based on race and ethnicity are summarized in Table 1. Participants were cisgender women (*Mage* = 20.90, *SD* = 1.96) who identified their primary race and ethnicity as Asian (32.7%), Black (33.9%), and Hispanic or Latina (33.3%). Among them, 76 (22.4%) were biracial or multiracial. The majority identified as straight or heterosexual (68.1%), followed by bisexual (22.7%). In the later analysis, sexual identity was dichotomized as (0) heterosexual and (1) LGBTQIA+ groups given the relatively small sample sizes in certain categories. One third (33.9%) were high school graduates, and 27.7% had partially completed college. Most were financially secure, with only 8.6% indicating they “can’t make ends meet.” Participants perceived their social status as mid-range (M = 5.48, SD = 1.87) on a 1 to 10 scale comparison to “people like them” (Adler et al., 2000). They reported using social media 6.4 days per week (SD = 1.3) and spent an average of 6 hours daily (SD = 2.86). Participants’ friends, on average, were 21.86 years old (SD = 1.96), with most friends aged between 18 and 24.

**Table 1:** Descriptive Statistics for Demographic Items Based on Primary Race and Ethnicity

	Asian		Black		Hispanic/		Total			
			*n* = 115		Latina		*n* = 339			
	*n* = 111				*n* = 113					
	*n*	%	*n*	%	*n*	%	*n*	%	*χ*2 (df)	*P*
**Biracial/Multiracial**	13	11.71	23	23	40	35.4	76	22.42	18.65 (2)	< 0.001
**Sexual Identity**									19.84 (14)	0.135
Heterosexual	85	76.58	74	64.35	72	63.72	231	68.14		
Gay	1	0.90	0	0	0	0	1	0.29		
Lesbian	3	2.70	5	4.35	2	1.77	10	2.95		
Bisexual	18	16.22	29	25.22	30	26.55	77	22.71		
Queer	1	0.90	1	0.87	1	0.88	3	0.88		
Pansexual	0	0.00	5	4.36	4	3.54	9	2.65		
Asexual	3	2.70	0	0	1	0.87	4	1.18		
I am not sure	0	0.00	1	0.87	3	2.65	4	1.18		
**Education Level**									23.08 (12)	0.027
8^th^ grade or less	0	0	0	0	1	0.88	1	0.29		
Partial high school	2	1.80	3	2.61	2	1.77	7	2.06		
High school graduate	26	23.42	46	40	43	38.05	115	33.92		
Partial college	38	34.23	26	22.61	30	26.55	94	27.73		
(at least one year)										
Associate’s	8	7.21	14	12.17	19	16.81	41	12.09		
College degree										
Undergraduate	27	24.32	16	13.91	12	10.62	55	16.22		
College degree										
Graduate degree	10	9.01	10	8.70	6	5.31	26	7.67		
**Financial Security**									5.31 (4)	0.275
We can’t make ends meet	5	4.50	13	11.3	11	9.73	29	8.55		
We have just enough	62	55.86	66	57.39	68	60.18	196	57.82		
We are comfortable	44	39.64	36	31.3	34	30.09	114	33.63		
**Social Media Use per Week**									35.29 (12)	< 0.001
0 days per week	1	0.90	1	0.87	0	0	2	0.59		
1 day per week	0	0	0	0	0	0	0	0		
2 days per week	0	0	5	4.35	1	0.88	6	1.77		
3 days per week	2	1.80	2	1.74	7	6.19	11	3.24		
4 days per week	8	7.21	11	9.57	1	0.88	20	5.90		
5 days per week	3	2.70	9	7.83	4	3.54	16	4.72		
6 days per week	7	6.31	14	12.17	3	2.65	24	7.08		
7 days per week	90	81.08	73	63.48	97	85.84	260	76.7		
**Social Media Use per Day**									37.21 (20)	0.011
0	1	0.90	1	0.87	0	0.00	2	0.59		
1	6	5.41	2	1.74	4	3.54	12	3.54		
2	14	12.61	6	5.22	6	5.31	26	7.67		
3	15	13.51	9	7.83	11	9.73	35	10.32		
4	15	13.51	12	10.43	12	10.62	39	11.50		
5	23	20.72	15	13.04	17	15.04	55	16.22		
6	3	2.70	12	10.43	16	14.16	31	9.14		
7	3	2.70	11	9.57	14	12.39	28	8.26		
8	6	5.41	8	6.96	10	8.85	24	7.08		
9	1	0.90	7	6.09	2	1.77	10	2.95		
10 or more hours	24	21.62	32	27.83	21	18.58	77	22.71		
**Friend’s age**									6.21 (8)	0.62
14 or lower	0	0.00	0	0.00	1	0.88	1	0.29		
14–18 years old	15	13.51	16	13.91	16	14.16	47	13.86		
18–24 years old	85	76.58	79	68.70	75	66.37	239	70.50		
24–30 years old	6	5.41	10	8.70	11	9.73	27	7.96		
30 and upper	5	4.50	10	8.70	10	8.85	25	7.37		

Significant racial or ethnic differences emerged. This study included more biracial or multiracial Hispanic or Latina participants (35.4%) compared to Black (23.0%) and Asian (11.71%) participants. Asian women (65.7%) were more likely to have some college experience or a higher education level than Black (48.7%) and Hispanic or Latina (54%) women. Most participants used social media seven days a week but with varied rates (Asian women at 81.08%, Black women at 63.48%, and Hispanic or Latina women at 85.84%). Yet daily usage showed Black participants (27.8%) more frequently reporting 10+ hours on social media than Asian (21.6%) and Hispanic or Latina (18.6%) participants.

### Gendered Racial Discrimination

Means, SDs, and results of statistical comparison tests for exposure to personal and vicarious in-person and social media gendered racial discrimination are shown in Table 2. On average, participants reported they were “sometimes” exposed to discrimination in the past month. More than 90% endorsed “witnessed people saying mean or rude things in public” or on social media. The four subscales were positively associated with each other, *r*s = 0.55–0.80. Thus, four subscales were combined as one scale in further analysis. Black women reported significantly higher levels of exposure to discrimination than both Asian (*p* = 0.001) and Hispanic or Latina women (*p* = 0.002). Hispanic or Latina (*p* = 0.049) women also reported higher levels of exposure to discrimination than Asian women.

**Table 2:** Means, Standard Deviations, and Results of MANOVAs for the Exposure to Gendered Racial Discrimination, Friendship Communications, Mental Health, and Covariates by Race and Ethnicity

Items	Asian	Black	Hispanic/ Latina	Total		
	M (SD)	M (SD)	M (SD)	M (SD)	F	*p*
**Gendered Racial Discrimination**						
Individual In-Person Subscale (Range 0–4)	1.26 (0.82)	1.89 (1.00)	1.54 (0.85)	1.57 (0.93)	13.70	< 0.001
Vicarious In-Person Subscale (Range 0–4)	1.86 (0.94)	2.34 (1.03)	2.17 (0.93)	2.12 (0.98)	7.12	< 0.001
Individual Online Subscale (Range 0–4)	0.91 (0.92)	1.44 (1.23)	1.11 (1.04)	1.16 (1.19)	7.07	< 0.001
Vicarious Online Subscale (Range 0–4)	1.87 (1.11)	2.38 (1.15)	2.09 (1.14)	2.12 (1.15)	5.72	< 0.001
**Gendered Racism Co-rumination** (Range 0–3)	1.63 (0.51)	1.8 (0.61)	1.72 (0.53)	1.72 (0.56)	2.58	0.077
**Gendered Racial or ethnic Socialization**						
Pride and empowerment subscale (Range 1–4)	3.11 (0.73)	3.44 (0.63)	3.25 (0.68)	3.27 (0.69)	6.44	0.002
Oppression awareness subscale (Range 1–4)	2.97 (0.64)	3.18 (0.73)	3.07 (0.58)	3.07 (0.66)	3.18	0.043
**Friendship Intimacy and Support**						
Support subscale (Range 1–3)	2.19 (0.57)	2.30 (0.50)	2.28 (0.50)	2.26 (0.52)	1.64	0.20
Intimacy subscale (Range 1–3)	2.26 (0.56)	2.49 (0.53)	2.38 (0.55)	2.38 (0.55)	5.13	0.006
**Depressive Symptoms** (Range 0–27)	11.16 (5.99)	12.46 (7.76)	10.83 (6.22)	11.49 (6.73)	1.88	0.16
**Anxiety Symptoms** (Range 0–21)	9.28 (4.89)	10.28 (6.27)	10.48 (5.78)	10.02 (5.69)	1.43	0.24
**Substance Use as Coping Strategy** (Range = 5–30)	9.38 (5.59)	13.11 (7.18)	10.70 (6.56)	11.09 (6.65)	9.67	< 0.001
**Gendered Racial or ethnic Identity** (Range 1–7)						
Identity commitment	5.17 (1.01)	5.27 (1.27)	5.55 (0.92)	5.33 (1.09)	4.76	0.009
Identity exploration	4.97 (1.14)	4.90 (1.29)	4.99 (1.04)	4.96 (1.16)	3.69	0.82
Identity centrality	4.57 (0.96)	4.55 (1.20)	4.92 (0.62)	4.68 (1.02)	0.19	0.026

To further illuminate the nature of discrimination, respondents’ narrative descriptions of gendered racial stereotypes were analyzed. For the open-ended question, 289 (85.25%) participants provided valid responses and of those 125 (43.25%) described gendered racial stereotypes. Narratives revealed both shared and unique stereotypes. Across racial or ethnic groups sexualization was a prominent theme, portraying women of color as hypersexual and subject to unwarranted sexual attention. Asian women highlighted experiences of fetishization, e.g. “It is harder to be a Filipino woman in the world because people either see you as a fetish or they see you as not serious.” Black women reported similar experiences, with one participant noting, “Many people see our features before they see us. They see sex and a baby mother…all from a skin tone.” Hispanic or Latina women expressed frustration with the “spicy Latina” stereotype, that one participant described as “dehumanizing” and perpetuated by social media posts like, “I need me a 5'0 thick, spicy Latina with braces.” While sexualization manifested uniquely across groups, it remained a shared form of discrimination that reinforced systemic objectification of women of color.

Participants also described unique gendered racial stereotypes. Asian women noted being stereotyped as “poor drivers,” that one participant explained led to “more aggressive drivers getting upset with Asians.” Black women frequently encountered the “Angry Black Woman” stereotype, misrepresenting their emotions as excessive or hostile, as one participant explained, “We’re always seen as overreacting, so people don’t take us seriously.” Hispanic or Latina women faced assumptions about immigration status. One Venezuelan immigrant shared, “I have been phased out, treated as a foreigner, and subjected to stereotypes that undermine my dignity and identity.”

### Friendship Experiences

On average, participants spent half their time online in the past month with friends (M = 53.77, SD = 28.63, Range = 0–100). They also spent between 43.26 - 46.81% of the time discussing racial issues. There were no racial or ethnic differences in time spent online, F(10, 644) = 1.58, *p* = 0.11, Pillai’s Trace = 0.05.

Means, standard deviations, and results of statistical comparison tests for friendship communications are shown in Table 2. Participants reported co-ruminating about gendered racism with their close friends approximately “often” and discussing pride and empowerment and oppression awareness conversations “often” to “always”. For friendship quality, since none of the participants selected the “Never” option in the perceiving support subscale, and ≤ 5 participants in each racial or ethnic group selected the “Rarely” option for multiple items in both support and intimacy subscales, responses were recoded as 3-level factor ranged from 1 = “Sometimes or less”, 2 = “Often” and 3 = “Always”. Participants on average reported “often” to “always” perceiving support and intimacy with their friends, suggesting an overall high quality of friendships in the current sample.

Black women reported higher levels of intimacy with friends than their Asian counterparts (*p* = 0.004). They were significantly more frequently discussing pride and empowerment (*p* = 0.001) and oppression awareness (*p* = 0.03) topics with their friends than Asian women. No racial or ethnic differences were found for co-rumination.

### Mental Health and Covariates

Descriptive statistics and results of comparison tests for mental health indices are provided in Table 2. With a score > 10 as the cutoff point, 48.67% of participants were at moderate risk for depression, and 44.25% were at moderate risk for general anxiety disorder, neither yielded racial or ethnic differences. For substance use as a coping mechanism for gendered racism, 55.9% of Asian women and 48.7% of Hispanic or Latina women had never used substances, compared to 25.2% of Black women. Black women scored significantly higher than Asian women on substance use as coping, *p* = 0.001. On average participants indicated they “somewhat agree” in response to the racial or ethnic development measures. Hispanic or Latina women scored significantly higher than Asian women in identity centrality, *p* = 0.025, and higher than Asian and Black women in identity commitment, *p*s = 0.029 and 0.016.

### Correlations Between Discrimination and Friendship Experiences and Mental Health

Table 3 presents the correlation results across studied variables and demographics. Hypotheses on the association between discrimination and mental health were supported. There were significant associations between exposure to discrimination with depressive and anxiety symptoms and substance use as coping. Hypotheses on associations between friendship experiences and mental health were partially supported. Co-rumination and oppression awareness socialization were positively associated with depressive and anxiety symptoms but not with substance use as coping. Friendship intimacy and support and pride and empowerment socialization were unrelated to any mental health indices.

**Table 3:** Bivariate Correlations with Bonferroni Adjustment among Studied Scales and Selected Covariates

	1	2	3	4	5	6	7	8	9	10	11	12	13	14	15	16	17	18	19	20	21
1. Exposure to discrimination	1																				
2. Depressive symptoms	0.26^***^	1																			
3. Anxiety symptoms	0.21^***^	0.79^***^	1																		
4. Substance use as coping	0.19^***^	0.31^***^	0.26^***^	1																	
5. Co-rumination	0.41^***^	0.13^*^	0.17^***^	0	1																
6. Friendship intimacy	0.09	0.04	0.06	0.04	0.27^***^	1															
7. Friendship support	0.01	0.07	0.09	0.05	0.22^***^	0.56^***^	1														
8. Pride and empowerment socialization	0.21^***^	0.02	0.04	−0.09	0.42^***^	0.37^***^	0.26^***^	1													
9. Oppression awareness socialization	0.38^***^	0.16^***^	0.12^*^	0.04	0.41^***^	0.36^***^	0.30^***^	0.62^***^	1												
10. Identity centrality	0.07	0.01	0.07	−0.05	0.21^***^	0.18^***^	0.10	0.21^***^	0.13^*^	1											
11. Identity exploration	0.12^*^	−0.01	0.09	−0.11	0.31^***^	0.23^***^	0.20^***^	0.31^***^	0.22^***^	0.58^***^	1										
12. Identity commitment	−0.03	−0.07	0.08	−0.15^**^	0.20^***^	0.24^***^	0.18^***^	0.30^***^	0.14^*^	0.62^***^	0.82^***^	1									
13. Age	0.09	−0.01	0.05	0.1	−0.03	−0.03	0.03	0.02	0.02	0.01	0.03	−0.01	1								
14. Education level	0	−0.04	−0.06	0.01	−0.06	0.003	0.03	−0.09	−0.01	−0.08	0.07	0	0.45^***^	1							
15. Financial insecurity	−0.03	0.21^***^	0.23^***^	0.17	0.01	−0.08	−0.08	−0.02	0.03	−0.01	0.02	−0.03	0.03	−0.02	1						
16. Self-reported social status	−0.03	−0.25^***^	−0.22^***^	−0.13^*^	0.05	0.06	0.05	0.004	−0.05	−0.02	0.02	0.01	0.01	0.11	0.45^***^	1					
17. Sexual identity^a^	−0.04	0.12^*^	0.11	0.09	−0.04	0.06	0.05	0.08	0.03	−0.04	0.13^*^	0.13^*^	−0.12^*^	−0.09	0.07	0.03	1				
18. Time online discussing racism	0.23^***^	−0.02	−0.03	0	0.27^***^	0.08	0.05	0.09	0.10	0.18^***^	0.05	0.11	−0.01	0.05	0.06	0.18^***^	−0.05	1			
19. Time online empowerment	0.19^***^	0.04	0.04	0.01	0.26^***^	0.11	0.06	0.03	0.10	0.15^*^	0.03	0.08	−0.01	0.01	0.01	0.12^*^	−0.07	0.64^***^	1		
20. Time online oppression awareness	0.18^***^	0.02	0.03	0.01	0.23^***^	0.06	0	0.10	0.09	0.14^*^	0.09	0.10	0.01	0.05	0.04	0.16^***^	−0.05	0.62^***^	0.85^***^	1	
21. Time online co-rumination	0.16^***^	0.01	0.01	0.01	0.23^***^	0.08	0.01	0.10	0.06	0.13^*^	0.04	0.05	−0.01	0.01	0	0.19^***^	−0.06	0.57^***^	0.80^***^	0.76^***^	1

^a^Sexual identity was recoded as a bivariate variable, 0 = Heterosexual, 1 = LGBTQIA + .

^*^p < 0.05, ^**^^*^p < 0.001.

### The Mediating Role of Gendered Racism Co-rumination

**Fig. 1:**
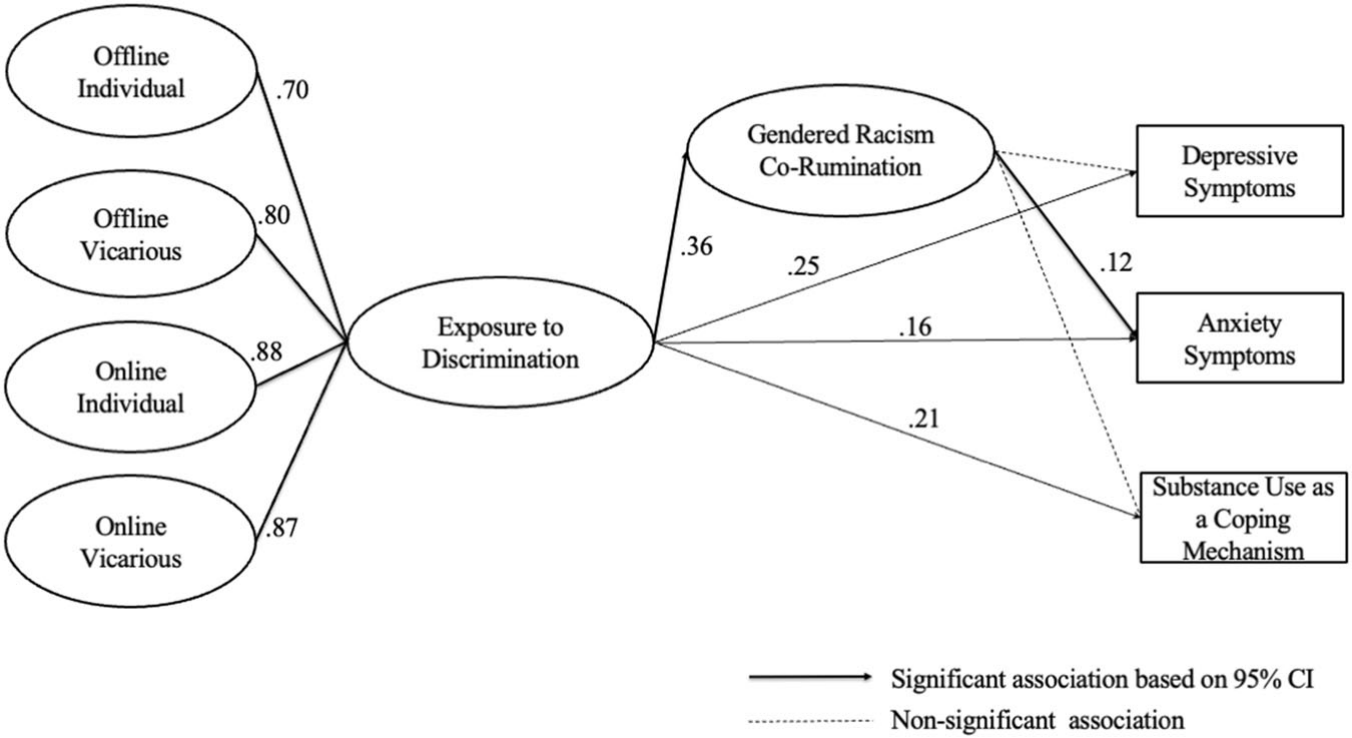
Results of the Model Predicting the Mediating Role of Gendered Racism Co-rumination in the Association between Exposure to Discrimination and Mental Health. Covariates including race and ethnicity, sexual identity, financial insecurity, self-reported social status, identity centrality and exploration, and time spent online discussing racial issues with friends, were omitted for presentation

Table 4 and Fig. 1 present the results of the model predicting the mediating role of gendered racism co-rumination in the association between exposure to discrimination and mental health. The latent factors included exposure to discrimination, represented by an overall latent factor estimated from 4 latent factors (each represented a subscale), and co-rumination. Covariates included race and ethnicity, with Black women as the reference group based on their highest levels of exposure to discrimination, sexual identity, financial insecurity, self-reported social status, REI exploration and centrality, and time spent online discussing racial issues with friends. Partially consistent with the mediation hypothesis, the indirect effect from exposure to discrimination to anxiety symptoms (*β* = 0.043, 95% CI [0.001, 0.091]) through co-rumination and the direct effect (*β* = 0.16, 95% CI [0.036, 0.25]) were both significant, indicating that gendered racism co-rumination with friends partially mediated the association between exposure to discrimination and anxiety symptoms. Contrary to expectations, only the direct effects of discrimination on depressive symptoms and substance use as coping were statistically significant (*β* = 0.25, 95% CI [0.14, 0.35] & *β* = 0.21, 95% CI [0.11, 0.30]), while the indirect effects were not (*β* = 0.021, 95% CI [−0.014, 0.066] & *β* = −0.034, 95% CI [−0.082, 0.00]), indicating that co-rumination failed to mediate the associations.

**Table 4:** Fit Indices for the Mediation and Moderated Mediation Structural Equation Modeling Results

Model	CFI	TLI	RMSEA	90% CI Lower	90% CI Upper
Mediating Role of Gendered Racism Co-rumination	0.95	0.95	0.041	0.038	0.044
Moderated Mediation Model with Pride and Empowerment Socialization as Moderator	0.90	0.89	0.041	0.037	0.044
Moderated Mediation Model with Friendship Intimacy as Moderator	0.94	0.94	0.039	0.036	0.042
Moderated Mediation Model with Friendship Support as Moderator	0.95	0.94	0.038	0.035	0.041
Sensitivity Analysis: Mediation Role of Depressive Symptoms	0.94	0.94	0.043	0.040	0.046
Sensitivity Analysis: Mediating Role of Anxiety Symptoms and Substance Use as Coping	0.94	0.94	0.044	0.042	0.047

### The Moderating Roles of Friendship Experiences

Before addressing the moderation hypothesis with latent moderation models, correlations (See Table 3) were examined. Exposure to gendered racial discrimination was positively associated with co-rumination and socialization but not with friendship intimacy and support. Friendship indices were all positively associated with each other. More specifically, intimacy and support (r = 0.56) and the two types of socialization (*r* = 0.61) were moderately to strongly associated. Thus, these four scales were included as separate models in later analysis.

Four models were tested to assess whether friendship experiences moderated the associations between exposure to discrimination and mental health. Given that latent interactions were estimated using algorithms that do not use a standard likelihood function, making traditional fit statistics unattainable, initial models included friendship intimacy, support, and socialization as predictors without interactions to assess model fit. All studied variables were latent factors. All models included the same set of covariates as those used in the mediation model. Results demonstrated good model fit (see Table 5 for model fit indices and parameter estimates). Discrimination yielded significant main effects on depressive and anxiety symptoms and substance use as coping across all models. Friendship experiences were unrelated to mental health in all models, with one exception, pride and empowerment socialization was negatively associated with substance use as coping, and perceived support was positively associated with depressive symptoms. However, no moderating effects of socialization or friendship quality were identified, the hypothesis was not supported.

**Table 5:** Results of Latent Moderation Analyses

Predictor	Depressive Symptoms (β [95% CI])	Anxiety Symptoms (β [95% CI])	Substance Use as Coping (β [95% CI])
**Pride and Empowerment Socialization**			
Exposure to Discrimination	**0.43 [0.25, 0.58]**	**0.33 [0.15, 0.50]**	**0.30 [0.19, 0.47]**
Pride and Empowerment	−0.03 [−0.20, 0.10]	−0.02 [−0.17, 0.10]	−**0.24 [**−**0.51**, −**0.12]**
Interaction	0.08 [−0.16, 0.32]	−0.03 [−0.32, 0.27]	−0.03 [−0.27, 0.15]
**Oppression Awareness Socialization**			
Exposure to Discrimination	**0.39 [0.22, 0.58]**	**0.34 [0.16, 0.50]**	**0.25 [0.15, 0.47]**
Oppression Awareness	0.12 [−0.05, 0.34]	−0.03 [−0.24, 0.20]	−0.01 [−0.29, 0.16]
Interaction	0.10 [−0.18, 0.40]	−0.05 [−0.23, 0.35]	0.20 [−0.14, 0.47]
**Friendship Intimacy**			
Exposure to Discrimination	**0.44 [0.25, 0.58]**	**0.33 [0.15, 0.50]**	**0.26 [0.19, 0.47]**
Friendship Intimacy	0.06 [−0.07, 0.18]	0.07 [−0.06, 0.25]	0.06 [−0.10, 0.17]
Interaction	−0.06 [−0.29, 0.13]	−0.05 [−0.30, 0.22]	0.03 [−0.35, 0.30]
**Friendship Support**			
Exposure to Discrimination	**0.44 [0.26, 0.58]**	**0.34 [0.14, 0.48]**	**0.26 [0.16, 0.41]**
Friendship Support	**0.17 [0.03, 0.31]**	0.15 [−0.01, 0.30]	0.15 [−0.02, 0.30]
Interaction	−0.05 [−0.21, 0.11]	−0.08 [−0.34, 0.22]	−0.03 [−0.34, 0.29]

Statistically significant results based on 95% CI are bolded. CFI = 0.90, TLI = 0.90, RMSEA = 0.048, 90% CI [0.045, 0.052] for pride and empowerment socialization; CFI = 0.91, TLI = 0.90, RMSEA = 0.052, 90% CI [0.047, 0.056] for oppression awareness socialization; CFI = 0.92, TLI = 0.91, RMSEA = 0.047, 90% CI [0.042, 0.051] for friendship intimacy; CFI = 0.92, TLI = 0.91, RMSEA = 0.048, 90% CI [0.043, 0.053] for friendship support.

### Moderated Mediation Models

In each model, the latent factors included exposure to discrimination, co-rumination, and the moderator. Initial testing of the four mediation models, incorporating friendship socialization, intimacy, and support as covariates for predicting mental health, demonstrated good model fit for pride and empowerment socialization and intimacy and support (see Table 4 for model fit indices). In contrast, the model for oppression awareness socialization showed poor fit (CFI = 0.87, TLI = 0.86), precluding further moderation testing. Subsequently, three moderated mediation models were evaluated.

**Fig. 2:**
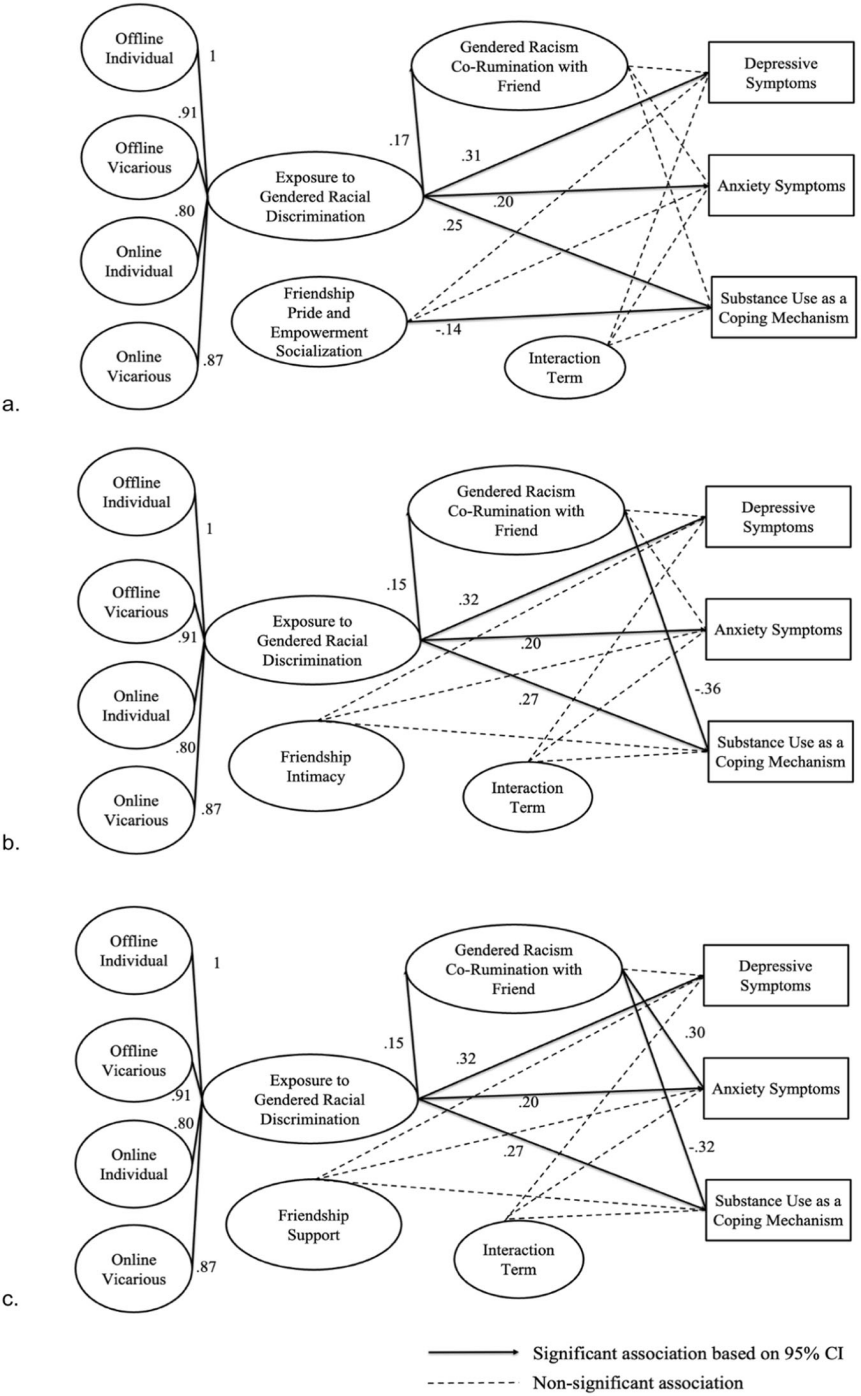
Results of the Model Predicting the Moderating Role of Friendship Experiences and Mediating Role of Gendered Racism Co-rumination in the Association between Exposure to Discrimination and Mental Health. **a**. Friendship Pride and Empowerment Socialization as the moderator. **b**. Friendship Intimacy as the moderator. **c**. Friendship Support as the moderator. Covariates for all models including race/ethnicity, sexual identity, financial insecurity, self-reported social status, identity centrality and exploration, and time spent online discussing racial issues with friends, were omitted for presentation

The model (Fig. 2a) examined the moderating role of pride and empowerment socialization in the associations across exposure to discrimination, co-rumination, and mental health symptoms did not yield a significant indirect effect from discrimination to depressive symptoms (*β* = 0.016, 95% CI [−0.09, 0.06]), anxiety symptoms (*β* = 0.047, 95% CI [−0.02, 0.1]), or substance use as coping (*β* = −0.025, 95% CI [−0.10, 0.02]) via co-rumination. Pride and empowerment socialization did not moderate the associations between discrimination and depressive (*β* = −0.06, 95% CI [−0.15, 0.22]) and anxiety symptoms (*β* = −0.03, 95% CI [−0.08, 0.15]), or substance use as coping (*β* = −0.008, 95% CI [−0.11, 0.09]). The direct effect of discrimination to mental health remained significant (*β* = 0.31, 95% CI [0.14, 0.45] for depressive symptoms, *β* = 0.20, 95% CI [0.09, 0.34] for anxiety, and *β* = 0.25, 95% CI [0.17, 0.36] for substance use as coping). There was a significant negative direct association between pride and empowerment socialization and substance use as coping (*β* = −0.14, 95% CI [−0.22, −0.03]).

The model (Fig. 2b) examined the moderating role of friendship intimacy in the associations across exposure to discrimination, co-rumination, and mental health symptoms also yielded significant direct effects of discrimination on mental health (β = 0.32, 95% CI [0.16, 0.42] for depressive symptoms, β = 0.20, 95% CI [0.06, 0.32] for anxiety, β = 0.27, and 95% CI [0.14, 0.36] for substance use as coping). The indirect effect from discrimination to anxiety via co-rumination was no longer significant (β = 0.041, 95% CI [−0.004, 0.089]), and there was an inconsistent indirect effect (Blalock, 1969; MacKinnon et al., 2000) for substance use as coping (β = −0.052, 95% CI [−0.12, −0.014]). No significant indirect effect was found for depressive symptoms (β = −0.003, 95% CI [−0.007, 0.028]). Friendship intimacy did not moderate the associations between discrimination and depressive symptoms (β = −0.047, 95% CI [−0.18, 0.08]), anxiety (β = −0.017, 95% CI [−0.15, 0.14]), or substance use as coping (β = 0.074, 95% CI [−0.06, 0.20]).

The model (Fig. 2c) for friendship support yielded similar results. The direct effect of discrimination on mental health remained significant (*β* = 0.32, 95% CI [0.17, 0.45] for depressive symptoms, *β* = 0.20, 95% CI [0.06, 0.32] for anxiety, *β* = 0.27, 95% CI [0.13, 0.36] for substance use). The indirect effect from discrimination to depressive symptoms (*β* = −0.003, 95% CI [−0.047, 0.044]) was not significant, but there was a positive indirect effect on anxiety via co-rumination (*β* = 0.045, 95% CI [0.002, 0.094]). Co-rumination emerged as an inconsistent mediator (MacKinnon et al., 2000) in the positive association between discrimination and substance use as coping (*β* = −0.047, 95% CI [−0.11, −0.009]). Friendship support did not moderate the associations between exposure to discrimination and depressive symptoms (*β* = −0.058, 95% CI [−0.15, 0.08]), anxiety symptoms (*β* = −0.033, 95% CI [−0.15, 0.09]), or substance use as coping (*β* = −0.008, 95% CI [−0.11, 0.13]). Overall, this hypothesis was not supported.

### Sensitivity Analysis

To assess the alternative relationship between co-rumination and mental health symptoms, an additional analysis was conducted examining whether depressive and anxiety symptoms and substance use as coping mediated the association between exposure to discrimination and co-rumination. Given the strong association between depressive and anxiety symptoms, two separate models were tested. Both models yielded good fit (See Table 4 for fit indices). The first model tested the mediating role of depressive symptoms. Results indicated that the indirect effect of discrimination on co-rumination through depressive symptoms (β = 0.005, 95% CI [−0.38, 0.45]) was not significant, while the direct effect of discrimination on co-rumination (β = 0.36, 95% CI [0.27, 0.68]) was significant. The second model tested the mediating role of anxiety symptoms and substance use as coping. Neither the indirect effect of discrimination on co-rumination through anxiety symptoms (β = 0.02, 95% CI [−0.13, 0.45]) nor through substance use as coping (β = −0.01, 95% CI [−0.03, 0.008]) was statistically significant. However, the direct effect of discrimination on co-rumination was significant (β = 0.35, 95% CI [0.11, 0.60]).

## Discussion

Exposure to discrimination rooted in the intersecting societal influences of sexism and racism is an important, yet understudied, aspect of the lived experience of young women of color (Bowleg, 2012; Cole, 2009). The current study found that women across different racial or ethnic groups encountered gendered racial discrimination, underscoring the universality of these experiences due to shared systemic patriarchy and endemic racism. The most prevalent type of discrimination experienced by participants (around 95%) was witnessing mean or rude comments about women of their race or ethnicity online. Moreover, spending more time with friends online was associated with greater exposure to discrimination, supporting prior observations that social media amplifies exposure to discriminatory content (Are, 2020). Although online discrimination occurs more frequently, reports of online and in-person forms of discrimination were highly correlated suggesting that exposure to gendered racism is a consistent part of racial related adversity in the lives of women of color (Chae et al., 2021). The quantitative measure of gendered racial discrimination used in the current study was further supported by qualitative insights that highlight both shared and distinct gendered racial stereotypes when women were asked to discuss stereotypes of women of their race or ethnicity. The richness of the participants’ responses underscores the profound impact of gendered racism on women of color, revealing both common and unique experiences across racial and ethnic groups.

Consistent with the minority stress theory (Frost & Meyer, 2023) and prior research (Portillo et al., 2022; Tao & Fisher, 2022), the present study supported the predicted relationship between young adult women of color’s exposure to gendered racial discrimination and mental health. Although high-quality friendships characterized by intimacy and support have been found to be a protective factor against identity threatening stressors among young adults (Davis & High, 2019), the influence of friendships in the context of gendered racism has not been examined. The current study contributes to this small yet growing literature by examining how friendship communications specifically focused on gendered racism impact the associations between exposure to gendered racial discrimination and mental health among young adult women of color. Overall, exposure to in-person and online discrimination was associated with more co-rumination about gendered racism. In turn, co-rumination was positively associated with anxiety symptoms, supporting research indicating repeated and excessive discussion about gendered racial discrimination increases mental distress (Spendelow et al., 2017; Stone & Veksler, 2022). However, co-rumination did not mediate the association between discrimination and depressive symptoms. The differential association between co-rumination and anxiety and depressive symptoms may stem from the nature of co-rumination, which involves prolonged and emotionally intense discussions of shared discriminatory experiences. While these discussions may initially provide validation and social connection, their repetitive focus on distress can intensify the worry and vigilance central to anxiety symptoms rather than the despair associated with depressive symptoms (American Psychiatric Association, 2013). Co-rumination and substance use as a coping mechanism were not associated. This suggests that these two behaviors may serve distinct functions for how women of color respond to discrimination. Co-rumination may help young women articulate and understand their experiences. In contrast, although the social context of substance use was not assessed in this study, it may operate as an alternative coping mechanism, offering temporary relief from distress independent of the social processing provided by co-rumination.

Alternative analyses tested whether mental health symptoms mediated the association between discrimination and co-rumination. In contrast to the hypothesis driven analyses demonstrating that co-rumination mediated the association between discrimination and anxiety, none of the mediation pathways in the alternative analyses were significant, indicating that the connection between discrimination and co-rumination may not be driven by psychopathological factors such as depressive or anxiety symptoms. Instead, co-rumination may arise from the social need to process and validate shared discriminatory experiences with trusted friends. Together with the main findings, these results highlight the unique role of co-rumination as an emotion-focused coping mechanism, particularly in amplifying anxiety by perpetuating hypervigilance and worry about discriminatory experiences.

The present study predicted that friendship socialization would be directly associated with mental health risk and either buffer or exacerbate the associations among exposure to discrimination, co-rumination, and mental health. However, these hypothesized relationships were only partially supported. Contrary to prior research (Ahn et al., 2022), pride and empowerment socialization were unrelated to depressive and anxiety symptoms. However, when accounting for exposure to discrimination, higher levels of pride and empowerment socialization were associated with less substance use as a means of coping with gendered racism. It is possible that such messaging may enhance self-esteem in young women of color, thereby reducing their immediate impulse to use substances and increasing their search for healthier coping strategies (Su et al., 2020). As predicted, oppression awareness messaging was positively associated with depressive symptoms. However, the anticipated association between oppression awareness and anxiety was not supported. This discrepancy may indicate that discussions emphasizing awareness of systemic oppression are more closely tied to feelings of hopelessness and resignation associated with depressive symptoms, rather than the uncertainty and vigilance associated with anxiety (American Psychiatric Association, 2013). The lack of association between oppression awareness socialization and substance use as coping with gendered racism suggests participants might be using alternative, more constructive responses to systemic oppression.

Contrary to expectation, friendship intimacy and support were unrelated to mental health. This finding is inconsistent with data identifying same race and ethnicity friendship quality as a buffer against a more general measure of identity threatening stressors (Davis & High, 2019). In contrast, when accounting for discrimination, social support was positively associated with depressive symptoms, suggesting that individuals experiencing higher levels of depressive symptoms are more likely to rely on their social networks for support. Unlike the gendered racism specific communications in the current study, neither the more general friendship intimacy nor support were related to exposure to discrimination. These findings suggest that while these friendship qualities are important, they may not serve as buffers against the mental health risks posed by gendered racism.

### Strengths, Limitations, and Future Directions

The current study extends understanding of the multifaceted nature of friendships among women of color and how friendship conversations focused on women’s exposure to gendered racism are associated with mental health among Asian, Black, and Hispanic or Latina young adult women. To our knowledge, this is the first study to examine how co-rumination against gendered racism, gendered racial socialization, and friendship quality impact associations between exposure to discrimination and mental health. However, sample size limitations must be acknowledged, preventing this study from conducting multigroup SEM analysis to examine racial and ethnic differences in research hypotheses. Black and Hispanic or Latina women in our study reported higher levels of exposure to discrimination and Black women reported higher levels of friendship intimacy. Future study should increase sample size to examine the shared and unique patterns across exposure to discrimination, friendships, and mental health among women across different racial or ethnic groups. The recruitment of participants with same-gender and same-race or ethnicity close friends who are comfortable discussing racial issues resulted in a sample biased towards those with higher levels of friendship quality. Additionally, framing the study to focus on a friend with whom participants discuss race may have influenced their choice of friend, potentially prioritizing a friend most engaged in racial discussions rather than their closest or most supportive friend. Focusing on a single close friend rather than a broader friend circle may only partially represent the influence of real-life friendship networks on the association between gendered racial discrimination and mental health. Relatedly, discussions regarding gendered racism may not be limited to same-race friendships. Future studies should consider examining the influence of a range of friendship types and employing social network analysis to explore these broader friendship structures. Moreover, this study only accounted for the mode of communications (percent of time communicating in-person versus online) as a covariate, such that differences between in-person and online friendships were not explored. Future research should examine how in-person and online friendships impact women of color’s communications and related mental health outcomes. In addition, this study did not focus on the in-group versus out-group context of exposure to discrimination, which remains an important avenue for future research. The cross-sectional nature of this study limits the causal inferences of the results and understanding of changes over time. Directions of significant associations between exposure to discrimination, friendship experiences, and mental health found in the current study may differ from the interpretation. Future research will benefit from longitudinal studies to examine the trajectories of discrimination experiences and how they impact mental health and social dynamics over time. This approach will play a critical role in developing more effective interventions and support systems responsive to the changing needs of young women of color as they navigate through different life stages. The current study incorporates qualitative insights from participants’ responses to an open-ended question, illuminating both shared and unique gendered racial stereotypes across women of color from different racial or ethnic groups. However, the qualitative data in the study did not capture the full complexity of these stereotypes or their direct impact on discrimination. Future research should use more structured methods to examine how specific stereotypes shape the experiences of gendered racism across different racial or ethnic groups.

## Conclusion

In-person and social media gendered racial discrimination represents a critical stressor for young women of color, yet its association with mental health and the role of close friendships in such association remain underexplored. Addressing this gap, this study examined how co-rumination against gendered racism, gendered racial socialization, and friendship quality mediate or moderate the association between exposure to discrimination and mental health symptoms among Asian, Black, and Hispanic or Latina young women. Findings revealed that co-rumination partially explained the association between discrimination and anxiety symptoms, emphasizing the emotional burdens of prolonged discussions about discrimination. While pride and empowerment messaging within friendships showed negative association with substance use as a coping mechanism, oppression awareness messaging was positively associated with depressive and anxiety symptoms, reflecting the psychological toll of recognizing systemic inequities. This study contributes to the growing body of research highlighting the importance of taking an intersectional approach to understanding how multiple systemic biases can increase mental health symptoms among socially marginalized groups. The complexity of friendship dynamics and associated mental health among young women of color navigating gendered racism during a unique developmental period opens up a fruitful avenue for future investigation. By identifying the associations across discrimination, friendship communication, and mental health, this study underscores the importance of fostering resilience against gendered racism within young women of color’s friendships and contributes to the broader understanding of intersectional stressors in early adulthood.

## Data Availability

The datasets generated and/or analyzed during the current study are not publicly available but are available from the corresponding author on reasonable request.

## References

[CR1] Adler, N. E., Epel, E. S., Castellazzo, G., & Ickovics, J. R. (2000). Relationship of subjective and objective social status with psychological and physiological functioning: Preliminary data in healthy, White women. *Health Psychology*, *19*(6), 586. 11129362 10.1037//0278-6133.19.6.586

[CR2] Ahn, L. H., Keum, B. T., Meizys, G. M., Choudry, A., Gomes, M. A., & Wang, L. (2022). Second-generation Asian American women’s gendered racial socialization. *Journal of Counseling Psychology*, *69*, 129–145. 10.1037/cou0000575. 34242043 10.1037/cou0000575

[CR3] American Psychiatric Association. (2013). *Diagnostic and statistical manual of mental disorders: DSM-*5 (Vol. 5, Issue 5). American psychiatric association Washington, DC. 10.1176/appi.books.9780890425596.

[CR4] Angelini, F., Marino, C., & Gini, G. (2022). Friendship quality in adolescence: The role of social media features, online social support and e-motions. *Current Psychology*. 10.1007/s12144-022-03564-3. 10.1007/s12144-022-03564-3 PMC9465130 36118141

[CR5] Are, C. (2020). How Instagram’s algorithm is censoring women and vulnerable users but helping online abusers. *Feminist Media Studies*, *20*(5), 741–744. 10.1080/14680777.2020.1783805.

[CR6] Arnett, J. J. (2000). Emerging adulthood. A theory of development from the late teens through the twenties. *The American Psychologist*, *55*(5), 469–480. 10842426

[CR7] Ayón, C. (2018). Latino Immigrant Family Socialization Scale: Development and Validation of a Multidimensional Ethnic–Racial Socialization Measurement. *Social Work*, *63*(3), 222–233. 10.1093/sw/swy016. 29701823 10.1093/sw/swy016

[CR8] Blalock, H. M. (1969). *Theory construction: From verbal to mathematical formulations*. Prentice-Hall Englewood Cliffs, NJ.

[CR9] Bowleg, L. (2012). The Problem With the Phrase Women and Minorities: Intersectionality—an Important Theoretical Framework for Public Health. *American Journal of Public Health*, *102*(7), 1267–1273. 10.2105/AJPH.2012.300750. 22594719 10.2105/AJPH.2012.300750 PMC3477987

[CR10] Brown, D. L., Blackmon, S., Rosnick, C. B., Griffin-Fennell, F. D., & White-Johnson, R. L. (2017). Initial Development of a Gendered-Racial Socialization Scale for African American College Women. *Sex Roles*, *77*(3), 178–193. 10.1007/s11199-016-0707-x.

[CR11] Cénat, J. M., Darius, W. P., Dalexis, R. D., Kogan, C. S., Guerrier, M., & Ndengeyingoma, A. (2024). Perceived racial discrimination, internalized racism, social support, and self-esteem among Black individuals in Canada: A moderated mediation model. *Cultural Diversity & Ethnic Minority Psychology*, *30*(1), 118–129. 10.1037/cdp0000542. 35420837 10.1037/cdp0000542

[CR12] Chae, D. H., Yip, T., Martz, C. D., Chung, K., Richeson, J. A., Hajat, A., Curtis, D. S., Rogers, L. O., & LaVeist, T. A. (2021). Vicarious racism and vigilance during the COVID-19 pandemic: Mental health implications among Asian and Black Americans. *Public Health Reports*, 00333549211018675. 10.1177/00333549211018675 PMC8203039 34034574

[CR13] Cole, E. R. (2009). Intersectionality and research in psychology. *American Psychologist*, *64*(3), 170–180. 10.1037/a0014564. 19348518 10.1037/a0014564

[CR14] Collins, P. H., & Bilge, S. (2020). *Intersectionality* (2nd edition). Polity.

[CR15] Crenshaw, K. (1989). Demarginalizing the intersection of race and sex: A black feminist critique of antidiscrimination doctrine, feminist theory and antiracist politics. *U. Chi. Legal f*., 139.

[CR16] Cyrus, K., & Morrison, C. (2019, December 11). *The ‘Q’ in LGBTQ: Queer/Questioning*. American Psychiatric Association. https://www.psychiatry.org:443/news-room/apa-blogs/the-q-in-lgbtq-queer-questioning.

[CR17] Davis, S. M., & High, A. C. (2019). Widening the gap: Support gaps in same race versus different race female friendship dyads. *Journal of Social and Personal Relationships*, *36*(1), 187–213. 10.1177/0265407517722245.

[CR18] ElTohamy, A., Hyun, S., Rastogi, R., Finneas Wong, G. T., Kim, G. S., Chae, D. H., Hahm, H. “Chris, & Liu, C. H. (2024). Effect of vicarious discrimination on race-based stress symptoms among Asian American young adults during the COVID-19 pandemic. *Psychological Trauma: Theory, Research, Practice, and Policy*, *16*(2), 217–224. 10.1037/tra0001480. 37227832 10.1037/tra0001480 PMC10674031

[CR19] Essed, P. (1991). *Understanding Everyday Racism: An Interdisciplinary Theory*. SAGE.

[CR20] Fisher, C. B., Tao, X., & Jaber, R. (in press). The Adolescent Discrimination Distress Index: Contributions and Challenges in the Measurement of Ethnic and Racial Discrimination. In *The Cambridge Handbook of Ethnic/Racial Discrimination and Youth Development*.

[CR21] Fisher, C. B., Wallace, S. A., & Fenton, R. E. (2000). Discrimination distress during adolescence. *Journal of Youth and Adolescence*, *29*(6), 679–695.

[CR22] Forbes, N., Yang, L. C., & Lim, S. (2023). Intersectional discrimination and its impact on Asian American women’s mental health: A mixed-methods scoping review. *Frontiers in Public Health*, 11. 10.3389/fpubh.2023.993396. 10.3389/fpubh.2023.993396 PMC10008964 36923035

[CR23] Frost, D. M., & Meyer, I. H. (2023). Minority stress theory: Application, critique, and continued relevance. *Current Opinion in Psychology*, *51*, 101579 10.1016/j.copsyc.2023.101579. 37270877 10.1016/j.copsyc.2023.101579 PMC10712335

[CR24] Gelles-Watnick, R. (2024, January 31). Americans’ Use of Mobile Technology and Home Broadband. *Pew Research Center*. https://www.pewresearch.org/internet/2024/01/31/americans-use-of-mobile-technology-and-home-broadband/.

[CR25] Gottfried, J. (2024, January 31). Americans’ Social Media Use. *Pew Research Center*. https://www.pewresearch.org/internet/2024/01/31/americans-social-media-use/.

[CR26] Hacker, D. S., Haywood, J. E., Maduro, R. S., Mason, T. B., Derlega, V. J., Harrison, S. B., & Socha, T. J. (2016). Reactions of African American Students to the George Zimmerman Trial: Co-Rumination and Thought Intrusions as Mediators. *Journal of Loss and Trauma*, *21*(6), 507–521. 10.1080/15325024.2016.1157405.

[CR27] Hu, L., & Bentler, P. M. (1999). Cutoff criteria for fit indexes in covariance structure analysis: Conventional criteria versus new alternatives. *Structural Equation Modeling: A Multidisciplinary Journal*, *6*(1), 1–55. 10.1080/10705519909540118.

[CR28] Hughes, D., & Johnson, D. (2001). Correlates in Children’s Experiences of Parents’ Racial Socialization Behaviors. *Journal of Marriage and Family*, *63*(4), 981–995. 10.1111/j.1741-3737.2001.00981.x.

[CR29] Jones, M. K., & Briones, M. (2022). The impact of marianismo on the association between intersectional discrimination and depressive symptoms among latina women: A profile analysis. *Journal of Latinx Psychology*, *10*, 304–321. 10.1037/lat0000211.

[CR30] Jones, M. K., Leath, S., Settles, I. H., Doty, D., & Conner, K. (2022). Gendered racism and depression among Black women: Examining the roles of social support and identity. *Cultural Diversity and Ethnic Minority Psychology*, *28*, 39–48. 10.1037/cdp0000486. 34291983 10.1037/cdp0000486

[CR31] Jones, M. S., Womack, V., Jérémie-Brink, G., & Dickens, D. D. (2021). Gendered racism and mental health among young adult US Black women: The moderating roles of gendered racial identity centrality and identity shifting. *Sex Roles: A Journal of Research*, *85*(3–4), 221–231. 10.1007/s11199-020-01214-1.

[CR32] Juang, L. P., Shen, Y., Kim, S. Y., & Wang, Y. (2016). Development of an Asian American Parental Racial–Ethnic Socialization Scale. *Cultural Diversity & Ethnic Minority Psychology*, *22*(3), 417–431. 10.1037/cdp0000083. 26866519 10.1037/cdp0000083 PMC7870365

[CR33] Klein, A., & Moosbrugger, H. (2000). Maximum likelihood estimation of latent interaction effects with the LMS method. *Psychometrika*, *65*(4), 457–474. 10.1007/BF02296338.

[CR34] Kroenke, K., Spitzer, R. L., & Williams, J. B. (2001). The PHQ-9: Validity of a brief depression severity measure. *Journal of General Internal Medicine*, *16*(9), 606–613. 10.1046/j.1525-1497.2001.016009606.x. PubMed. 11556941 10.1046/j.1525-1497.2001.016009606.x PMC1495268

[CR35] Le, T. P., & Iwamoto, D. K. (2019). A longitudinal investigation of racial discrimination, drinking to cope, and alcohol-related problems among underage Asian American college students. *Psychology of Addictive Behaviors*, *33*(6), 520–528. 10.1037/adb0000501. 31414850 10.1037/adb0000501

[CR36] Lewis, J. A. (2018). From modern sexism to gender microaggressions: Understanding contemporary forms of sexism and their influence on diverse women. In *APA handbook of the psychology of women: History, theory, and battlegrounds, Vol. 1* (pp. 381–397). American Psychological Association. 10.1037/0000059-019.

[CR72] MacCallum, R. C., Browne, M. W., & Sugawara, H. M. (1996). Power analysis and determination of sample size for covariance structure modeling. *Psychological methods*, *1*, 130–149.

[CR37] MacKinnon, D. P., Krull, J. L., & Lockwood, C. M. (2000). Equivalence of the Mediation, Confounding and Suppression Effect. *Prevention Science: The Official Journal of the Society for Prevention Research*, *1*(4), 173. 11523746 10.1023/a:1026595011371 PMC2819361

[CR38] Macqueen, K., McLellan-Lemal, E., Kay, K., Milstein, B., & Cdc, A. (1998). Codebook Development for Team-Based Qualitative Analysis. *Cultural Anthropology*, *10*, 31–36. 10.1177/1525822X980100020301.

[CR39] Martz, C. D., Allen, A. M., Fuller-Rowell, T. E., Spears, E. C., Lim, S. S., Drenkard, C., Chung, K., Hunter, E. A., & Chae, D. H. (2019). Vicarious Racism Stress and Disease Activity: The Black Women’s Experiences Living with Lupus (BeWELL) Study. *Journal of Racial and Ethnic Health Disparities*, *6*(5), 1044–1051. 10.1007/s40615-019-00606-8. 31215018 10.1007/s40615-019-00606-8 PMC7302115

[CR40] Mendelson, M. J., & Aboud, F. E. (1999). Measuring friendship quality in late adolescents and young adults: McGill Friendship Questionnaires. *Canadian Journal of Behavioural Science / Revue Canadienne Des Sciences Du Comportement*, *31*, 130–132. 10.1037/h0087080.

[CR41] Moody, A. T., Lewis, J. A., & Owens, G. P. (2022). Gendered Racism, Coping, and Traumatic Stress Among Black Women: The Moderating Roles of the Strong Black Woman Schema and Womanist Attitudes. *Psychology of Women Quarterly*, 03616843221143752. 10.1177/03616843221143752.

[CR42] Navaneetham, P., & Kanth, B. (2022). Effects of Personal Relationships on Physical and Mental Health among Young Adults- A Scoping Review. *The Open Psychology Journal*, 15(1). 10.2174/18743501-v15-e2208180.

[CR43] Neblett, E. W., Bernard, D. L., & Banks, K. H. (2016). The Moderating Roles of Gender and Socioeconomic Status in the Association Between Racial Discrimination and Psychological Adjustment. *Cognitive and Behavioral Practice*, *23*(3), 385–397. 10.1016/j.cbpra.2016.05.002.

[CR44] Nesi, J., Choukas-Bradley, S., & Prinstein, M. J. (2018). Transformation of Adolescent Peer Relations in the Social Media Context: Part 1—A Theoretical Framework and Application to Dyadic Peer Relationships. *Clinical Child and Family Psychology Review*, *21*(3), 267–294. 10.1007/s10567-018-0261-x. 29627907 10.1007/s10567-018-0261-x PMC6435354

[CR45] Nolen-Hoeksema, S., Wisco, B. E., & Lyubomirsky, S. (2008). Rethinking rumination. *Perspectives on Psychological Science*, *3*(5), 400–424. 26158958 10.1111/j.1745-6924.2008.00088.x

[CR46] Paley, A. (2022). *The Trevor Project National Survey*. https://www.TheTrevorProject.org/survey-2021/.

[CR47] Pearce, E., Machin, A., & Dunbar, R. I. M. (2021). Sex Differences in Intimacy Levels in Best Friendships and Romantic Partnerships. *Adaptive Human Behavior and Physiology*, *7*(1), 1–16. 10.1007/s40750-020-00155-z.

[CR48] Phinney, J. S. (1992). The Multigroup Ethnic Identity Measure: A New Scale for Use with Diverse Groups. *Journal of Adolescent Research*, *7*(2), 156–176. 10.1177/074355489272003.

[CR49] Portillo, N. L., Grapin, S. L., Reyes-Portillo, J. A., & Masia Warner, C. (2022). Online discrimination and mental health outcomes: The moderating roles of ethnic identity and immigrant generation among Latinx young adults. *Journal of Latinx Psychology*, *10*, 322–339. 10.1037/lat0000212.

[CR50] Rose, A. J. (2002). Co-rumination in the friendships of girls and boys. *Child Development*, *73*(6), 1830–1843. 10.1111/1467-8624.00509. 12487497 10.1111/1467-8624.00509

[CR51] Sellers, R. M., Rowley, S. A., Chavous, T. M., Shelton, J. N., & Smith, M. A. (1997). Multidimensional Inventory of Black Identity: A preliminary investigation of reliability and constuct validity. *Journal of Personality and Social Psychology*, *73*(4), 805.

[CR52] Sobieraj, S. (2018). Bitch, slut, skank, cunt: Patterned resistance to women’s visibility in digital publics. *Information, Communication & Society*, *21*(11), 1700–1714. 10.1080/1369118X.2017.1348535.

[CR53] Spendelow, J. S., Simonds, L. M., & Avery, R. E. (2017). The Relationship between Co-rumination and Internalizing Problems: A Systematic Review and Meta-analysis. *Clinical Psychology & Psychotherapy*, *24*(2), 512–527. 10.1002/cpp.2023. 27215974 10.1002/cpp.2023

[CR54] Spitzer, R. L., Kroenke, K., Williams, J. B., & Löwe, B. (2006). A brief measure for assessing generalized anxiety disorder: The GAD-7. *Archives of Internal Medicine*, *166*(10), 1092–1097. 10.1001/archinte.166.10.1092. 16717171 10.1001/archinte.166.10.1092

[CR55] Stevenson, H. C. (1994). Validation of the Scale of Racial Socialization for African American Adolescents: Steps toward Multidimensionality. *Journal of Black Psychology*, *20*(4), 445–468. 10.1177/00957984940204005.

[CR56] Stokes, M. N., Hope, E. C., Cryer-Coupet, Q. R., & Elliot, E. (2020). Black Girl Blues: The Roles of Racial Socialization, Gendered Racial Socialization, and Racial Identity on Depressive Symptoms among Black Girls. *Journal of Youth and Adolescence*, *49*(11), 2175–2189. 10.1007/s10964-020-01317-8. 32955702 10.1007/s10964-020-01317-8

[CR57] Stone, L. B., & Veksler, A. E. (2022). Stop talking about it already! Co-ruminating and social media focused on COVID-19 was associated with heightened state anxiety, depressive symptoms, and perceived changes in health anxiety during Spring 2020. *BMC Psychology*, *10*(1), 22 10.1186/s40359-022-00734-7. 35130965 10.1186/s40359-022-00734-7 PMC8819886

[CR58] Su, J., Kuo, S. I.-C., Derlan, C. L., Hagiwara, N., Guy, M. C., & Dick, D. M. (2020). Racial discrimination and alcohol problems among African American young adults: Examining the moderating effects of racial socialization by parents and friends. *Cultural Diversity and Ethnic Minority Psychology*, *26*(2), 260–270. 10.1037/cdp0000294. APA PsycInfo. 31328948 10.1037/cdp0000294 PMC6980251

[CR59] Szymanski, D. M., & Lewis, J. A. (2016). Gendered racism, coping, identity centrality, and African American college women’s psychological distress. *Psychology of Women Quarterly*, *40*(2), 229–243. 10.1177/0361684315616113.

[CR60] Tao, X., & Fisher, C. (2023). Associations Among Web-Based Civic Engagement and Discrimination, Web-Based Social Support, and Mental Health and Substance Use Risk Among LGBT Youth: Cross-Sectional Survey Study. *Journal of Medical Internet Research*, *25*(1), e46604 10.2196/46604. 37358882 10.2196/46604 PMC10337473

[CR61] Tao, X., & Fisher, C. B. (2022). Exposure to Social Media Racial Discrimination and Mental Health among Adolescents of Color. *Journal of Youth and Adolescence*, *51*(1), 30–44. 10.1007/s10964-021-01514-z. 34686952 10.1007/s10964-021-01514-z PMC8535107

[CR62] Turner, R. J. (1999). Social support and coping. In *A handbook for the study of mental health: Social contexts, theories, and systems* (pp. 198–210). Cambridge University Press.

[CR63] Tynes, B. M., Rose, C. A., & Williams, D. R. (2010). The development and validation of the online victimization scale for adolescents. *Cyberpsychology: Journal of Psychosocial Research on Cyberspace*, 4(2). Article 2. https://cyberpsychology.eu/article/view/4237.

[CR64] Veenstra, R., & Laninga-Wijnen, L. (2021). *The Prominence of Peer Interactions, Relationships, and Networks in Adolescence and Early Adulthood*. OSF. 10.31235/osf.io/s57zm.

[CR65] Watkins, E. R., & Roberts, H. (2020). Reflecting on rumination: Consequences, causes, mechanisms and treatment of rumination. *Behaviour Research and Therapy*, *127*, 103573 10.1016/j.brat.2020.103573. 32087393 10.1016/j.brat.2020.103573

[CR66] Wei, M., Alvarez, A. N., Ku, T.-Y., Russell, D. W., & Bonett, D. G. (2010). Development and validation of a Coping with Discrimination Scale: Factor structure, reliability, and validity. *Journal of Counseling Psychology*, *57*(3), 328–344. 10.1037/a0019969. 21133583 10.1037/a0019969

[CR67] Williams, D. R., Yu, Yan, null, Jackson, J. S., & Anderson, N. B. (1997). Racial Differences in Physical and Mental Health: Socio-economic Status, Stress and Discrimination. *Journal of Health Psychology*, *2*(3), 335–351. 10.1177/135910539700200305. 22013026 10.1177/135910539700200305

[CR68] Winchester, L. B., Jones, S. C. T., Allen, K., Hope, E., & Cryer-Coupet, Q. R. (2022). Let’s talk: The impact of gendered racial socialization on Black adolescent girls’ mental health. *Cultural Diversity and Ethnic Minority Psychology*, *28*, 171–181. 10.1037/cdp0000484. 34410753 10.1037/cdp0000484

[CR69] Yip, T. (2018). Ethnic/Racial Identity—A Double-Edged Sword? Associations With Discrimination and Psychological Outcomes. *Current Directions in Psychological Science*, *27*(3), 170–175. 10.1177/0963721417739348. 30581253 10.1177/0963721417739348 PMC6301037

[CR70] Yip, T., Wang, Y., Mootoo, C., & Mirpuri, S. (2019). Moderating the association between discrimination and adjustment: A meta-analysis of ethnic/racial identity. *Developmental Psychology*, *55*(6), 1274–1298. 10.1037/dev0000708. 30907605 10.1037/dev0000708 PMC6557142

[CR71] Zhou, Y., MacGeorge, E. L., & Myrick, J. G. (2020). Mental Health and Its Predictors during the Early Months of the COVID-19 Pandemic Experience in the United States. *International Journal of Environmental Research and Public Health*, *17*(17), 6315 10.3390/ijerph17176315. 32877985 10.3390/ijerph17176315 PMC7503583

